# Advances in the surface modification techniques of bone-related implants for last 10 years

**DOI:** 10.1093/rb/rbu007

**Published:** 2014-10-20

**Authors:** Zhi-Ye Qiu, Cen Chen, Xiu-Mei Wang, In-Seop Lee

**Affiliations:** ^1^Institute for Regenerative Medicine and Biomimetic Materials, School of Materials Science and Engineering, Tsinghua University, Beijing 100084, China, ^2^Beijing Allgens Medical Science and Technology Co., Ltd, Beijing 100176, China, ^3^Bio-X Center, School of Life Science, Zhejiang Sci-Tech University, Hangzhou 310018, China, and ^4^Institute of Natural Sciences, Yonsei University, Seoul 120-749, Korea

**Keywords:** surface modification, physicochemical coating, radiation grafting, plasma surface engineering, ion beam processing, surface patterning, bone-related materials

## Abstract

At the time of implanting bone-related implants into human body, a variety of biological responses to the material surface occur with respect to surface chemistry and physical state. The commonly used biomaterials (e.g. titanium and its alloy, Co–Cr alloy, stainless steel, polyetheretherketone, ultra-high molecular weight polyethylene and various calcium phosphates) have many drawbacks such as lack of biocompatibility and improper mechanical properties. As surface modification is very promising technology to overcome such problems, a variety of surface modification techniques have been being investigated. This review paper covers recent advances in surface modification techniques of bone-related materials including physicochemical coating, radiation grafting, plasma surface engineering, ion beam processing and surface patterning techniques. The contents are organized with different types of techniques to applicable materials, and typical examples are also described.

## Introduction

Biological responses to implants largely depend on the surface properties of biomaterials, such as surface chemistry and physical structure [1–5]. As implants are inserted in human body, a variety of acute or chronic responses occur at the biomaterial surface.

In most bone-related implants, sufficient mechanical strength is required [6, 7]. For example, an intervertebral fusion cage needs to possess high compressive strength and fine fatigue strength [8], and an artificial hip joint should resist wearing associated with friction between femur head and acetabular cup [6]. Therefore, metallic biomaterials, bioceramics and polymers with good mechanical properties were developed to meet such requirements. Besides having good mechanical properties, biocompatibilities of such materials are very important factors to be considered for the long-term success of implants, especially for metallic biomaterials as these materials are known as biotolerant [9–11].

**Figure 1.**
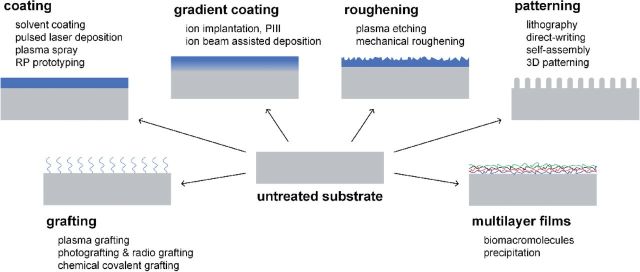
Schematic diagrams of surface modification techniques.

As for an example, Ti–6Al–4V and Co–Cr alloys have been commonly used for artificial joints, however, their wear debris produced by long-term friction would induce inflammatory responses and even result in aseptic loosening of the joint [12, 13]. Therefore, various surface modification techniques were developed to improve their tribological properties. Some commonly used surface modification techniques and their effects are shown in Fig. 1.

Biomaterial surface can be modified by these illustrated methods to overcome or reduce their inherent shortages or disadvantages. As for the requirements of better biomaterials, the cutting edge techniques were introduced to improve surface physical, chemical and biological properties of bone grafts, so as to meet the clinical requirements of bone defect substitution and repair. This review covers recent advances in coating, non-coating and patterning techniques for the surface modification of bone graft materials.

In this review article, contents are arranged with the clue of different types of the surface modification techniques. The commonly used substrate materials and typical examples are involved in each section. It is important to note that laser is a widely used light beam source for many modification techniques, since it is able to rapidly and effectively induce physical and/or chemical changes (e.g. roughness [14], deformation [15], polymerization and grafting) on various biomaterial surfaces. Since many of surface modification techniques involve the use of laser, the applications of laser technique are described in relevant sections rather than a separate one.

## Surface Modification with Coating Layer

Coating techniques are simple and intuitive approaches to obtain a modified surface. A variety of conventional physical and chemical coating methods (e.g. solvent evaporation, plasma spraying, and physical/chemical vapor deposition (CVD)) have been industrialized, while novel approaches involving recently developed and developing techniques are continuously coming forth. What will be introduced in this section are state-of-the-art physical and chemical methods for creating functional coatings on different biomaterial surfaces.

### Rapid prototyping

Rapid prototyping (RP) comprises a series of techniques using three-dimensional computer-aided design (CAD) data to quickly fabricate a model or duplicate a same part. Some of RP techniques were used to construct coating for biomaterials, especially for metallic biomaterials.

Laser engineered net shaping (LENS) is an additive RP manufacturing technique that uses a focused, high-energy laser beam to melt metallic powders directly injected to the focused laser beam spot to form a new layer. Balla *et al.* [16] coated titanium with tantalum by using a LENS process to obtain better osseointegration property. Graded Co–Cr–Mo alloy coating was also successfully created on porous Ti6Al4V surface by LENS to obtain a high hardness interface [17]. Besides fabricating metallic coating, LENS is able to prepare ceramic coating. Roy *et al.* [18] successfully fabricated calcium phosphate coating on titanium without phase transition of the ceramic coating.

### Pulsed laser deposition

Pulsed laser deposition (PLD), a physical vapor deposition (PVD) method, is popular for fabricating calcium phosphate coating on metallic substrate, since it is able to stoichiometrically transfer material from target to substrate and could obtain a ultra-thin coating layer (thickness of several atoms) [19]. Although PLD has been introduced to the surface modification of biomaterials for nearly 20 years, this technique is continuously developing in the field. For example, in its recent development, PLD was used to fabricate calcium phosphate coating on porous Ti6Al4V substrate produced by selective laser melting (SLM, one of RP techniques) [20]; water-assisted PLD was developed to improve coating-substrate binding strength [21]. PLD has also been introduced to the surface coating on polymers in recent years. Prosecka *et al.* [22] fabricated thin layer of hydroxyapatite (HA) on caprolactone/polyvinyl alcohol composite nanofibers. Besides calcium phosphate coating, bioceramic coating composed of akermanite (Ca_2_MgSi_2_O_7_) was successfully created on both non-biodegradable polysulfone and bioresorbable polylactic acid (PLA) surface by PLD [23].

### Ion beam-assisted deposition

Ion beam-assisted deposition (IBAD), which is also called ‘ion beam enhanced deposition’ (IBED) is a vacuum deposition surface modification technique that combines PVD and ion implantation (described in the section ‘Ion implantation and plasma immersion ion implantation’). In the IBAD, an ion beam bombardment is continuous throughout the process to clean substrate surface prior to the deposition and control depositing film properties during the deposition. A significant advantage of IBAD is that such technique is able to create a gradual transition layer mixed with substrate material and depositing material between the substrate and the deposited film, thereby the coating adheres strongly to the substrate.

**Figure 2.**
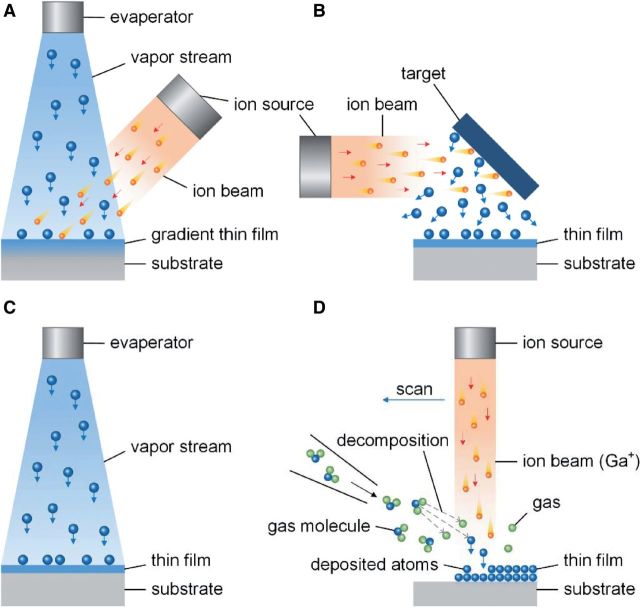
Schematic diagrams of ion beam surface modification methods (A: IBAD, B: IBSD, C: IBD and D: IBID).

It is important to distinguish IBAD from some other surface modification techniques that also use ion beam and have similar names, including ion beam deposition (IBD), ion beam induced deposition (IBID) and ion beam sputtering deposition (IBSD). IBD is a direct beam deposition (DBD) process that directly applies an ionized particle beam onto substrate surface to fabricate thin film [24]. A significant difference between IBD and ion implantation is that the ionized particle beam in the IBD has low energy, and the particles arrive at substrate surface with a ‘soft landing’ [24]. IBID is a CVD technique that uses focused ion beam (usually Ga^+^ ion beam) to decompose gaseous molecules and deposit non-volatile component onto substrate surface [25]. IBSD is a PVD process that an ion beam bombards a target and ejects particles in atomic scale from the target to form thin film on nearby substrate surface [26]. Schematic diagrams of these ion beam surface modification methods are illustrated in Fig. 2. Each of IBD, IBID and IBSD can be utilized for creating thin film coating on material surface, however, they are hardly to create gradual transition between the substrate and the deposited film as IBAD, thus obtaining relatively lower adhesive strength.

IBAD has been used for the surface modification of biomaterials for decades and is still in development. As a typical application, Cui *et al.* [27] fabricated HA coating on Ti–6Al–4V substrate with an atomic intermixed coating/substrate interface by IBAD. The deposition was performed by a multifunctional IBAD system. Before the deposition, the substrate surfaces were cleaned by Ar^+^ ion beam bombardment. Then, a composite target containing HA and tricalcium phosphate was sputtered by Ar^+^ ion beam to form the coating on the substrate, which was simultaneously bombarded by another energetic Ar^+^ ion beam. In the deposition process, the bombardment energy of Ar^+^ ion beam was relatively higher at first to produce atomic intermixed layer of the coating and the substrate, and then the bombardment energy was reduced to increase the thickness of the coating and reinforce the compactness. During the deposition, the temperature of the substrate was below 100°C, which did not affect the substrate. The adhesive strength of the coating fabricated by IBAD was tested to be nearly twice to that prepared by IBSD with the same processing environment. Chen *et al.* [28, 29] prepared calcium phosphate thin film coating on pure titanium by using IBAD and further created biomimetic apatite precipitation layers by immersing the coating in Dulbecco’s phosphate buffered saline solutions containing calcium chloride, as well as biomolecules to modulate precipitation processes and enhance bioactivities.

IBAD was also applicable for the fabrication of metallic, bioceramic and composite thin film coating for many varieties of biomaterials for bone graft (e.g. titanium, stainless steel and ultra-high molecular weight polyethylene [UHMWPE]) [30–33]. A drawback for IBAD is that it is a line-of-sight modification technique, so it is difficult for IBAD to treat an irregular surface with non-line-of-sight regions.

### Plasma coating

Plasma is a state of matter that is partially or fully ionized, and contains charged particles of free ions, electrons, radicals, as well as neutral particles of atoms and molecules. Plasma could be divided into thermal (high-temperature/hot/equilibrium) one and nonthermal (low-temperature/cold/nonequilibrium) one. The thermal plasma is nearly fully ionized, and electrons and heavy particles have the same temperature. The temperature required to generate thermal plasma is typically ranging from 4000 to 20 000 K [34]. Such high temperature is destructive for biomaterials, especially for those polymers. For nonthermal plasma, only a small fraction of the gas molecules are ionized, and ions and neutrals are at a much lower temperature (may as low as room temperature), although the temperature of electrons could reach several thousand degrees Celsius. The plasma used for the surface modification of biomaterials is the nonthermal one, which can be generated by different sources, including corona discharge, dielectric barrier discharges, radio frequency discharges and so on [35].

Plasma surface engineering is a series of economic and effective approaches for the surface modification of biomaterials and has been applied to commercialized products [36]. Plasma treatments can be used to modify material surfaces via different processes, including etching (or ablation), sputtering, polymerization, grafting and spray [35]. Wherein, plasma spray, plasma sputtering and plasma polymerization could be used to produce coating on a surface. Other plasma processes for the surface modification will be attributed to non-coating techniques.

#### Plasma spray

Plasma spray is a coating process that sprays melted or partially melted coating material onto substrate surface, and this technique has been applied to commercially available bone implants. The energy and temperature of the plasma environment of plasma spray are relatively higher than plasma surface engineering techniques descripted above. Due to its high operating temperature, plasma spray is usually applied to fabricate various coatings on metallic biomaterials, such as apatite and its derivatives coating [37–39], calcium silicate coating [40, 41], bioglass coating [42–44], zirconia coating [45], titanium coating [46] and composite coating [47, 48]. Moreover, bioceramic coatings have been successfully fabricated on polymer substrate by using plasma spraying, for example, HA coating on polyetheretherketone (PEEK) or carbon fiber-reinforced polyamide 12 [49, 50], and titanium coating on carbon fiber-reinforced PEEK [51]. However, the influences of the high-energy plasma on the polymer substrate were not discussed by these studies.

**Figure 3.**
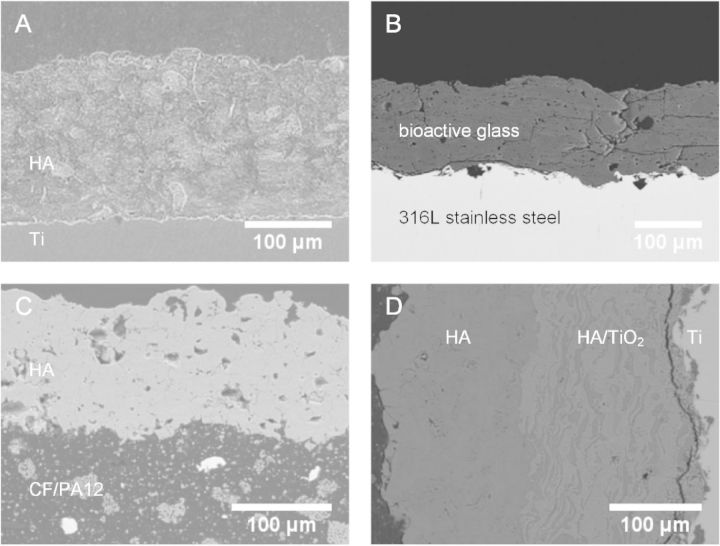
Cross-sectional micrographs of coatings on various substrates prepared via plasma spray. (A: HA coating on titanium substrate (Reproduced with permission from Ref. [52], Copyright 2007 Elsevier Ltd.), B: 31SiO_2_-56CaO-2MgO-11P_2_O_5_ bioactive glass coating on 316L stainless steel substrate (Reproduced with permission from Ref. [53], Copyright 2013 Elsevier Ltd.), C: HA coating on carbon fibers/polyamide 12 (CF/PA12) composite substrate (Reproduced with permission from Ref. [49], Copyright 2005 John Wiley & Sons, Inc.), D: HA and HA/TiO_2_ coating on titanium substrate (Reproduced with permission from Ref. [54], Copyright 2004 Elsevier Ltd.)).

Figure 3 shows cross-sectional micrographs of coatings on various biomaterials that have been used for bone implants. The thickness of plasma sprayed coatings was usually more than 100 μm, and the interface could be clearly observed between the substrate and the coating. Therefore, the interface binding strength produced by plasma spray is relatively lower than those by IBAD with a gradient transition layer.

#### Plasma polymerization

Plasma polymerization is a process that ionizes monomer gas into plasma state and induces radical polymerization to create polymer coating on a substrate, so as to enhance corrosion resistance of metallic biomaterials or improve biocompatibility and bioactivity of relatively inert materials [55–57]. For example, Lewis *et al.* [55] fabricated fluorocarbon film on 316L stainless steel, and the corrosion rate was significantly decreased compared with those uncoated; Liu *et al.* [58] employed plasma polymerization to modify surfaces via generating different functional groups (amine, carboxyl, methyl and hydroxyl) and found that the plasma polymerization of allylamine on the surface promoted osteogenic differentiation of human adipose-derive stem cells [58].

## Surface Modification with Grafting and Implantation

Coating approaches can effectively modify surface properties for bone implants. However, these coating techniques create isolation layer between the material and surrounding organisms, thus cutting off the interaction between them. Therefore, many advantageous properties of the substrates become useless for surrounding organisms after being coated. Moreover, most of those coatings are physically bound to the substrate, and the binding strength is limited.

Besides coating, there are many other techniques partially modify surface physical and/or chemical properties by, for example, grafting molecules on a surface, or injecting ions into superficial layer of a substrate. In this section, those surface modification techniques without forming coating are summarized as ‘non-coating’ methods, and several surface grafting methods and ion implantation techniques for the surface modification of bone implant materials will be introduced. Furthermore, these non-coating techniques do not modify topographic features in either micro- or nano-scale.

### Chemical covalent bonding

**Figure 4.**
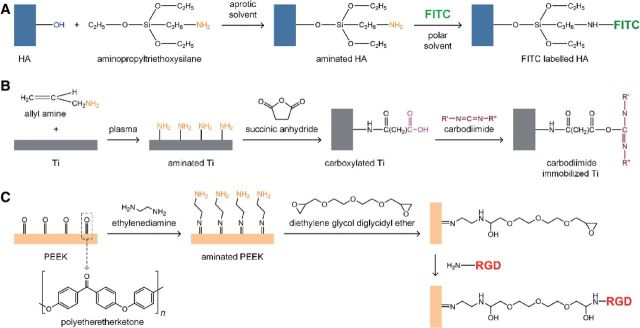
Chemical covalent bonding processes on different biomaterials. (A: silanization on calcium phosphate bioceramic [59, 60], B: carbodiimide immobilization on titanium metal [61] and C: polypeptide grafting on biopolymer [62]).

The use of functional groups on material surface to form covalent bond between the substrate and the coating is a classical approach for constructing chemical coating. The reaction is specific and binding effect is stable. Figure 4 shows some illustrative examples for chemical covalent bonding processes on biomaterials.

Silanization is a low-cost and effective covalent coating method to modify material surface that are rich in hydroxyl groups, such as HA, bioglass, titania and many other metal oxide surfaces. There are many types of commercially available silane coupling agents, which are easy to react with hydroxylated surface and introduce active groups (e.g. amino group and carboxyl group) to the surface. Figure 4A takes HA as the example to illustrate chemical structure of the modified surface. Silanized surface can easily be modified by further grafting. Zhang *et al.* labeled nanometer HA with fluorescein isothiocyanate (FITC) by modifying HA with 3-aminopropyltriethoxysilane (AMPTES), and then grafting FITC via reaction with the amino group [59, 60]. Although the silanization is simple and effective, the reaction conditions such as concentration of the silane and reaction time must be carefully controlled to prevent from forming thick polymerized silane network on the surface. Otherwise, the bond between silane and the surface can also subject to hydrolysis in some conditions [63].

### Photografting and radiation grafting

Chemical grafting has been widely used to obtain stable surface modification results for biomaterials. Active groups (e.g. –OH, –COOH and –NH_2_) exposed to the surface are necessary to acquire high chemical reactivity for the grafting. It is difficult to perform chemical grafting on the surface of those bioinert materials, since there are only a few or no active groups exposed to their molecular surface.

However, many relatively inert materials are being used as bone implants. In order to conduct grafting on the surface of these biomaterials, extra energy must be introduced to the grafting reaction. As the name suggests, photografting and radiation grafting make use of radiations, including UV radiation (photografting), gamma radiation and high-energy electron beam. The radiation breaks chemical bonds on material surface to be grafted, and form free radicals. The reactive surface will be then exposed to monomers to initiate surface graft polymerization [64].

The use of photografting and radiation grafting in the field of biomaterials is focused on surface modification of polymers, especially those hydrophobic and bioinert. Various materials commonly used in the preparation of bone substitutes, such as PEEK, UHMWPE and some biodegradable polymers, have been investigated to modify physical and chemical properties, as well as improve biocompatibility and osteointegration by radiation grafting and photografting [65, 66]. Photografting by UV radiation was used to improve the tribological performance of UHMWPE [66], enhance hydrophilicity of PEEK and biodegradable polymers [67, 68] and adjust biodegradation rate of PLA [69]. Gamma radiation grafting was reported to graft poly(*N*-isopropylacrylamide), a polymer had low critical solution temperature onto the surface of polystyrene Petri dish to control attachment and detachment of cells [70]; Cho *et al.* [71] used gamma radiation for the surface modification of UHMWPE by the graft polymerization of methyl methacrylate (MMA) monomer, so as to improve interfacial strength with poly(methyl methacrylate) (PMMA) bone cement. Electron beam grafting was used to enhance the hydrophilicity of PMMA [72], and improve biocompatibility of bioinert polymer [73].

### Plasma etching and grafting

Plasma can not only be used to prepare coatings on biomaterials, but is also be able to conduct various non-coating surface modification processes, for example, plasma etching and plasma grafting.

Plasma etching modifies a surface by shooting a high-speed stream of plasma onto the substrate. Plasma etching is helpful to improve surface activity for bioinert polymers, with less influence on surface topography than chemical etching process [74].

Plasma grafting are used to modify surface chemical properties of biomaterials by grafting active groups on the surface, for example, plasma grafting of zinc oxide onto polypropylene to obtain an antibacterial surface [75].

### Ion implantation and plasma immersion ion implantation

#### Ion implantation

**Figure 5.**
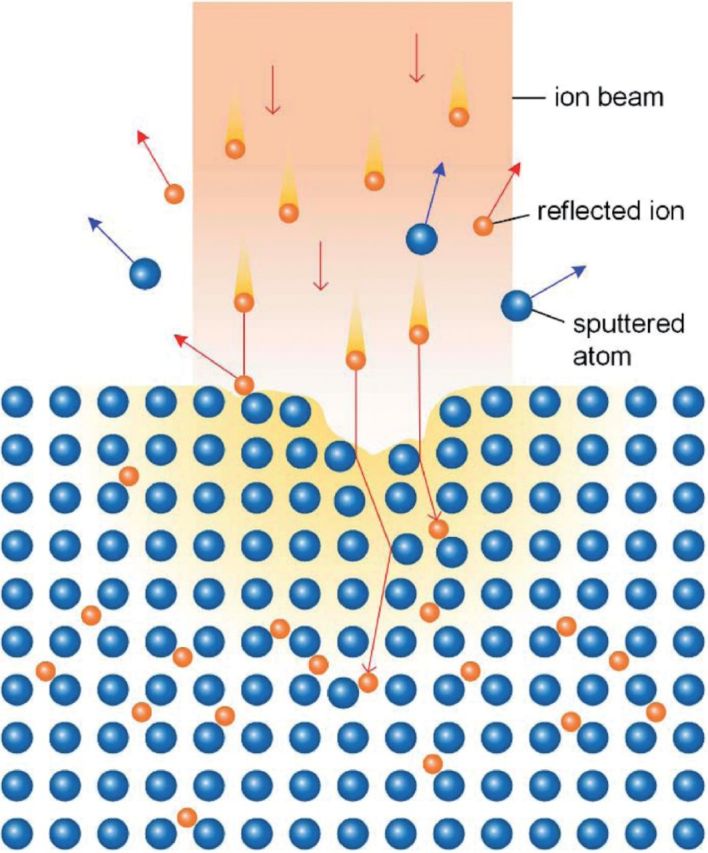
Schematic diagram of ion implantation process.

Ion implantation is a physical surface modification process that injects accelerated high-energy ions into the surface of a material to modify its physicochemical and biological properties (Fig. 5). Almost every atom within the periodic table is available for ion implantation. For a biomaterial surface, ion implantation could be used to enhance corrosion resistance, reduce wear debris, regulate hardness and improve biocompatibility and bioactivities. For example, iridium was implanted into Ti–6Al–4V surface to enhance corrosion resistance [76]; nitrogen ion implantation into Ti–6Al–4V and UHMWPE can reduce surface wear [77]; ion implantation of silver into surfaces of 317L stainless steel, titanium, and Ti–Al–Nb alloy increased their anti-bacterial natures [78]; graphene could attain a good cytocompatibility by NH_2_ ion implantation [79]. Ion implantation has also been used to modify surface properties of polymers for biomedical applications [80, 81].

Ion implantation has many advantages: material of the substrate is unrestrictive, since the high-energy ions are forcibly injected into substrate surface; the implanted ions dispersed within a certain depth of substrate surface without forming a new layer, avoiding drawbacks (e.g. cracking and detachment) of traditional coatings; low operating temperature (sometimes at room temperature) did not affect the substrate material. However, traditional ion implantation is a line-of-sight processing technique, and is thus not suitable for the treatment of those bone implants with complex shape and internal structure, for example, a hip joint prosthesis with complex curved surface [82].

#### Plasma immersion ion implantation

Plasma immersion ion implantation (PIII) was invented by Prof. J. Conrad in 1980s [83], and overcame the limitation of line-of-sight processing of traditional ion implantation [84, 85]. In a PIII process, a workpiece to be modified on the surface is immersed in a plasma atmosphere and applied with a direct current (DC) or a high voltage pulsed DC, ions from the plasma are accelerated in the electric field of plasma sheath surrounding and perpendicular everywhere to workpiece surface, and finally implanted into the surface [86]. Therefore, PIII is able to modify complex surface or even inner surface of a material [85], which is of significance to those bone grafts with irregular shape and structure. In early 1990s, plasma immersion ion implantation and deposition (PIII&D) was then developed based on PIII by Brown *et al.* [87, 88] to build up a thin film with an atomically mixed interface with the substrate, so as to expand pure ion implantation technique to a hybrid coating technique.

PIII and PIII&D have now been applied to the surface modification of various biomaterials, including metals and polymers, in terms of surface mechanical properties, biocompatibility, bioactivity, antibacterial activity and so on [89, 90]. For example: nitrogen, oxygen and hydrogen implanted metallic materials (e.g. Ti–6Al–4V and stainless steel) and polymers (e.g. UHMWPE and PEEK) used for orthopedic implants showed better wear performance than those untreated [91–94]; corrosion resistance of alloys containing hazardous elements for the health (e.g. Ni–Ti alloy) could be enhanced by using oxygen, nitrogen, carbon or acetylene PIII and PIII&D [95–98]; for those bioinert materials for medical use (e.g. titanium, polytetrafluoroethylene and PEEK), it has been reported that biocompatibility and bioactivities (e.g. osteogenic) are able to be significantly improved by applying PIII and PIII&D using oxygen, hydrogen, water, calcium or zinc plasma on the surface modification [99–103]; antibacterial surface for biomaterials could be fabricated by introducing silver (Ag) or copper (Cu) element into the surface by using PIII and PIII&D [104–106].

## Surface Patterning of Biomaterials in Micro- and Nano-Scale

Studies on cell biology demonstrated that the topography of the extracellular matrix (ECM) could regulate stem cell behaviors and fate, such as cell growth and differentiation, via physical interactions with the cells. Such physical interactions are affected by some geometric cues in different scales, including molecular conformation, surface topography or roughness, fiber diameter and so on [107]. The bone tissue was proposed to be divided into as many as nine levels from molecules (e.g. collagen and HA) to a bone organ, wherein many levels have their specific patterns in sub-nano- or micro-scale. For example, array patterns of mineralized collagen fibrils in nano-scale, and material patterns of woven bone, parallel fibered bone and lamellar bone in micro-scale [108]. Therefore, behaviors and fate of osteocytes would be regulated by topographies of bone tissue in various scales.

Based on the regulation effects of topography on cell behavior and fate in natural tissues, material could be functionalized by modifying surface topography, for example, creating patterns. As early as 1911, Harrison [109] found the influence of topography on cell behaviors based on the observation of the relationship between the movements of embryonic cells and the material shapes in contact with the cells. As the development of nano- and micro-processing techniques, some of them have been employed to fabricate nano- and micro-patterns on biomaterial surfaces, and interactions between patterned biomaterial surfaces and cells were investigated.

Surface patterning techniques are widely used and are developing rapidly in the field of microelectronics. Recently advanced methods for surface patterning on biomaterials were partially derived from those in microelectronics industry [110]. In this section, methodologies for the fabrication of nano- and micro-patterns on material surfaces are summarized.

### Photolithography with mask

**Figure 6.**
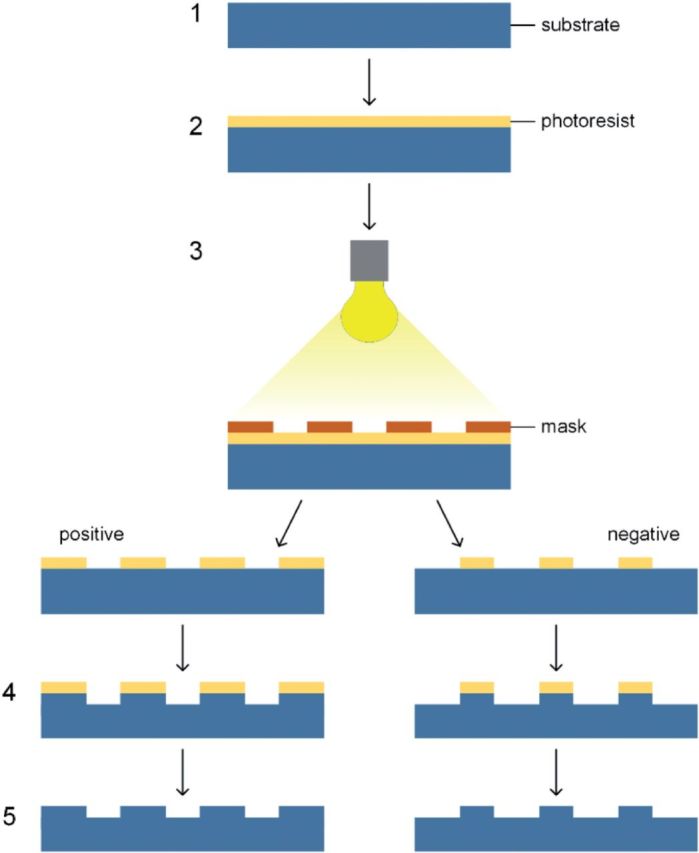
Schematic diagram of photolithography procedures.

Photolithography was the first surface patterning technique introduced to make patterns for controlling cell behaviors [111]. Another well-known application of photolithography in the field of biology is the fabrication of DNA arrays [112, 113]. A photolithography process commonly comprises following steps (as shown in Fig. 6): (i) prepare a clean and flat substrate; (ii) coat a light sensitive polymer (called photoresist) onto the substrate; (iii) expose the photoresist under a mask (usually quartz or metal) to form a desired pattern; (iv) transfer the pattern to the substrate by an etching process (development process) and (v) remove the photoresist. Wherein, the photoresist could be either a positive one that areas exposed to the light beam can be dissolved in the development process (illustrated as ‘positive’ one in Fig. 6), or a negative one works conversely (illustrated as ‘negative’ one in Fig. 6). Due to the optical diffraction of the focused light beam, the resolution of the pattern created by photolithography is restricted to about half of the wavelength of the light source, typically several hundred nanometers [114]. Actually, 1 µ would be the finest resolution for photolithography in practice. Therefore, photolithography is suitable to create patterns with the size comparable to a cell.

### Direct-write photolithography

Above classic photolithography with mask is an indirect approach for the fabrication of surface patterns. As the advance of photolithography, a direct-write mode without using mask has been developed via using a focused light beam to fabricate pattern directly on material surface [115–117]. In this maskless photolithography, laser is more commonly used as the light source to provide high-intensity light beam for the fabrication of surface patterns. In order to fabricate patterns, the light beam may induce two types of reactions on material surface: one is photochemical reaction for photoactive surfaces [118], the other is physical reactions involving ablation, melt or deformation of the substrate caused by the high energy of the laser [119, 120].

Some typical examples by using direct-write photolithography are: Pfleging *et al.* [121, 122] created micro-patterns on polystyrene surface by using laser photolithography combined with UV radiation to enhance L929 cells adhesion and protein adsorption; Rebollar *et al.* [123] fabricate submicro-patterns on polystyrene substrate to guide cell alignment and improve cell proliferation; Ahrem *et al.* [124] used pulsed laser to make 3D channels on bacterial cellulose hydrogels without chemical modifications or chemical strength lose, and observed inward migration of chondrocytes in the channels, as well as good matrix production and phenotypic stabilization.

### Electron beam lithography

With the similar principle and fabrication process to photolithography, electron beam lithography (EBL) is able to create nano-sized patterns on material surfaces, since the electron beam is considered as a de Broglie wave, the wavelength of the electron is much shorter than that of light beams [125]. For example, a typical electron beam provided by electron microscope is accelerated by 100 kV electric field, and the wavelength is 0.003 nm. EBL is used to make patterns on electron sensitive material surfaces, which would be crosslinked [126, 127], chemically transformed [128, 129], polymerized [130] and so on under the electron beam. For example, Idota *et al.* [130] used EBL to polymerized and grafted *N*-isopropylacrylamide onto a hydrophilic polyacrylamide-grafted glass surfaces to form patterns, so as to regulate cell attachment directions and detachment. In spite of the high spatial resolution in nano-scale, EBL has its own limitations, such as high cost and low throughput. Therefore, EBL is now more commonly investigated in laboratory, rather than industrialization applications.

Moreover, EBL was used to fabricate special patterns that gave extraordinary functions to material surfaces. Wang *et al.* [131] constructed a patterned surface with sub-micron sized polyethylene glycol (PEG) microgels by EBL technique, since the PEG microgels were non-adhesive to both cells and bacteria, the patterned surface would be non-adhesive to bacteria with comparable size to the PEG microgels, as well as did not affect adhesion and behaviors of normal cells. Such interesting effects were further investigated and many similar multi-functional patterned surfaces were developed [132, 133].

### Scanning probe lithography

Scanning probe lithography (SPL) is a direct-write method that moves a micro- or nano-stylus on material surface to mechanically ‘write’ patterns. SPL can be divided into two different types according to the patterning manners: the one is constructive that matters are transferred to the surface from the stylus (such as dip-pen nanolithography (DPN)); and the other one is destructive that the surface is deformed (such as nano-imprinting/engraving).

DPN uses a tip of atomic force microscope to create patterns by directly writing on the surface using a variety of molecular inks (solutions of molecules). In biomedical applications, DPN has been used to create patterns with polymers [134–136], biomolecules (including proteins [137], peptides [138], lipids [139, 140], enzymes [141] and DNA [142]), nano-particles [143–145], as well as living cells [146, 147], onto different substrates. Similar to other nano-patterning techniques mentioned above, the throughput of DPN is relatively low. To overcome such disadvantage, a high-throughput DPN was developed by parallelly operating a 2D probe arrays consisted of 55 000 tips [148].

Nano-imprinting and engraving uses a hard stylus to indent or scratch material surface to create patterns. Thus, the process is destructive to the surface. By using this process, nano-patterns were created on thin film (such as self-assembly monolayer) coated surface [149]. Moreover, nano-patterns of proteins were fabricated by grafting protein molecules onto the exposed substrate after the scratching [150, 151].

### Patterning with master

**Figure 7.**
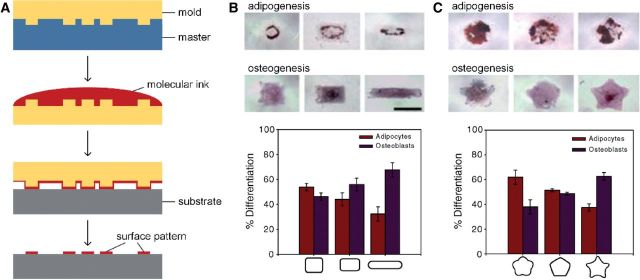
Operating steps of µCP and regulation effects of the patterns on cell fate (A: operating steps of µCP, B: regulation effects of the patterns on cell fate by varying aspect ratio [154] (Copyright 2010 National Academy of Sciences), C: regulation effects of different pattern shapes on cell fate [154] (Copyright 2010 National Academy of Sciences)).

Patterning with master uses a template with patterns to replicate patterns on a substrate, and the process is sometimes called ‘microcontact printing’ (µCP). Typically, as shown in Fig. 7A, a mold, typically made of elastomeric polymer (such as polydimethylsiloxane (PDMS)), is created according to the master, and then used to print patterns on the substrate with molecular inks [152]. In this patterning technique, the mold contacts with the substrate to transfer the patterns onto the surface by the molecular inks. As early as 1990s, Singhvi *et al.* [153] created patterns on a golden substrate with a PDMS stamp to control cell distribution and shape, and demonstrated such spatial restriction is helpful to maintain albumin secretion, which is an important physiological function of hepatocytes. Another example is the regulation of cell fate by patterning with master: Kilian *et al.* [154] created patterns with different shapes on a glass substrate coated by gold via µCP, after a period of culturing mesenchymal stem cells (MSCs) on the surface, the cells displayed different adipogenesis and osteogenesis profiles, indicated that modulation of cell shape was able to direct cell differentiation (Fig. 7B and C). Patterning with master has also been used to patterning proteins [155, 156], DNA [157] and cells [158, 159] for biomedical applications.

Another process is imprinting with master, which uses a template made of hard material for creating patterns on the substrate [160]. Imprinting with master makes mechanically deformation on material surface to form patterns. Therefore, the master for imprinting must be hard enough to fabricate patterns on a relatively soft substrate.

By combining imprinting with master and lithography, nanoimprint lithography (NIL) creates nano-patterns on a substrate by imprinting a mold with the nano-patterns into a resist coated on the substrate and subsequent etching process. NIL was firstly reported in 1996 by Prof. S. Chou and his coworkers [161]. They prepared a mold of nano-patterned silicon dioxide by EBL and etching, and then transferred the pattern to a PMMA resist coated on a silicon substrate by indenting to form patterned resist with different thickness, followed by anisotropic etching to finally transfer the pattern onto the substrate. Such NIL process used a nano-template to create pattern, rather than by using a stylus, brought about relatively higher throughput. NIL has been used to create nano-patterns on various substrates for biomedical applications [162–164], or fabricate polymers [165, 166], proteins [167–169] and DNA [170] patterns on the substrates.

### Self-assemble of molecules or nano-particles

Self-assemble is an energy-saving process to prepare patterns. During the self-assemble, intermolecular or inter-particle forces make molecules or nano-particles arranged in a regular pattern, in order to minimize total free energy of the entire surface. Molecules or nano-particles can self-assemble in an area ranging from nano-scale to micro-scale. Many types of materials were employed in self-assemble fabrication of patterns, such as block copolymers [171–173], nano-spheres [174, 175], nano-particles [176], biomolecules [177, 178] and so on.

### 3D patterning

Since the cells live and act in a 3D physiological environment in natural tissues and organs, 3D patterns on biomaterial surfaces would provide spatial architectures closed to physiological conditions and beneficial for tissue reconstruction and repair. As a result, 3D patterning technique is becoming an attractive research hotspot in the field of surface modification. In the present review paper, 3D patterning does not refer to a specific patterning techniques, many of those described in previous sections could be used to create 3D patterns via minor modifications or by combining with other techniques.

Two-photon lithography (TPL) and multiphoton lithography (MPL) are direct-write technique that is capable of creating 3D patterns on polymeric surfaces by laser beam. During a TPL or MPL process, two-photon or multiphoton absorption occurs at a photosensitive surface by attaining energy from the laser beam, thereby chemical reactions (usually polymerization) take place to form 3D patterns at the laser spot. For example, Nielson *et al.* [179] fabricated 3D patterns with bovine serum albumin by photocrosslinking using MPL on a coverglass according to high-resolution X-ray computed tomographic data. The 3D patterns were exactly replicated by using a dynamic mask and the resolution was as high as submicron (∼0.5 μm). Besides replicated patterns, crosslinked protein with unstrained structures in micro-scale could be fabricated using MPL [180]. For biomedical applications, MPL can be used to fabricate micro-patterns or 3D structures with proteins [179–181], hydrogels [182–184], bioabsorbable polymers [185, 186], gelatin [187, 188] and so on.

Interference lithography (IL), which is also called holographic lithography or interference holography, uses interference patterns formed by two coherent laser beams to build periodic 3D patterns. By adjusting parameters (e.g. phase, amplitude and polarization) of the coherent laser beams, features of the interference patterns could be modified. During an IL process, the interfering laser beams can be used to induce polymerization reactions to create 3D patterns on a substrate. The advantage of IL is that the patterning process is simple without using masks, and the throughput is relatively higher than other patterning processes. However, the alignment of the coherent beams is complex, and any changes for the patterns need to simultaneously adjust both beams. Although IL was developed in recent decade and relatively widely used in the field of microelectronics or optoelectronics [189–191], this technique has also been employed to fabricate patterns on materials for biomedical applications. For example, Prodanov *et al.* [163] produced nano-grooved surfaces with different features on titanium by laser IL, reactive ion etching and NIL techniques, animal implantation experiment demonstrated that the pattern with alternate 75 nm ridge and 225 nm groove achieved best early (4 weeks) osteointegration among all those patterned surfaces.

## Summary

Various surface modification techniques commonly used for bone-related implants are reviewed in this article. In practical terms, one approach would be chosen from various feasible candidates for surface modification according to the target effect and physiochemical properties of the substrate, also cost as an important factor. As comprehensive utilization of multiple methods is often required to fulfill the needs, it is necessary to fully understand the principles and effects of such many modification techniques, and their latest advances prior to the processing.
